# A systematic review of the reporting of Data Monitoring Committees' roles, interim analysis and early termination in pediatric clinical trials

**DOI:** 10.1186/1471-2431-9-77

**Published:** 2009-12-13

**Authors:** Ricardo M Fernandes, Johanna H van der Lee, Martin Offringa

**Affiliations:** 1 Departamento da Criança e da Família, Hospital de Santa Maria, Centro Hospitalar Lisboa Norte-EPE; and Laboratório de Farmacologia Clínica e Terapêutica, Instituto de Medicina Molecular, Faculdade de Medicina, Universidade de Lisboa, Portugal; 2 Department of Pediatric Clinical Epidemiology, Emma Children's Hospital, Academic Medical Centre, Amsterdam, the Netherlands

## Abstract

**Background:**

Decisions about interim analysis and early stopping of clinical trials, as based on recommendations of Data Monitoring Committees (DMCs), have far reaching consequences for the scientific validity and clinical impact of a trial. Our aim was to evaluate the frequency and quality of the reporting on DMC composition and roles, interim analysis and early termination in pediatric trials.

**Methods:**

We conducted a systematic review of randomized controlled clinical trials published from 2005 to 2007 in a sample of four general and four pediatric journals. We used full-text databases to identify trials which reported on DMCs, interim analysis or early termination, and included children or adolescents. Information was extracted on general trial characteristics, risk of bias, and a set of parameters regarding DMC composition and roles, interim analysis and early termination.

**Results:**

110 of the 648 pediatric trials in this sample (17%) reported on DMC or interim analysis or early stopping, and were included; 68 from general and 42 from pediatric journals. The presence of DMCs was reported in 89 of the 110 included trials (81%); 62 papers, including 46 of the 89 that reported on DMCs (52%), also presented information about interim analysis. No paper adequately reported all DMC parameters, and nine (15%) reported all interim analysis details. Of 32 trials which terminated early, 22 (69%) did not report predefined stopping guidelines and 15 (47%) did not provide information on statistical monitoring methods.

**Conclusions:**

Reporting on DMC composition and roles, on interim analysis results and on early termination of pediatric trials is incomplete and heterogeneous. We propose a minimal set of reporting parameters that will allow the reader to assess the validity of trial results.

## Background

Data Monitoring Committees (DMCs) are central to modern clinical trials. They play a critical role in safeguarding both the interests of study participants and the scientific integrity of randomized trials[1] This task is performed through interim monitoring of safety and efficacy outcome data, as well as vigilance over the conduct of the study and safety aspects/adverse events. One of the key activities of DMCs is to make recommendations to the sponsor or steering committee regarding the appropriateness of trial continuation. Thus, the recommendations of a DMC can influence the conclusion and interpretation of trial results and, indirectly, their implementation in clinical practice.

Experts and regulators have recently published recommendations for the appointment and operational procedures of DMCs [1-5] The increasing use and visibility of DMCs has fostered research into their activities[6] There is evidence of heterogeneity in their roles and policies [7-9] The wide range of interim monitoring methods that are available entails considerable variability in DMCs' risk-benefit evaluations[1,2,10] These issues are relevant as the analyses and decisions made by DMCs may be controversial and can influence study validity, particularly in the case of trials terminated early [11-14]

Assessing DMCs' recommendations requires adequate reporting of their activities and considerations. Previous studies have suggested that reporting is insufficient for various subsets of publications and types of trials[8,11,15-18] The CONSORT statement of reporting guidelines for clinical trials includes an item on interim analysis, but no details on a DMCs' activities and considerations are required[19]

There is consensus that DMCs should be a standard requirement for trials with vulnerable populations, such as children[1,4,5,7,20-22] In a recent review of pediatric trials performed until 2002, only 2% of the trials were reported to have a safety committee[23] Recent US and EU regulations that require research on medicines in children are expected to foster an increase in the number of pediatric drug trials in the coming years[24] This calls for the establishment of standards for trial conduct and reporting in this field, in which the need for transparency of the roles of DMCs is an important issue[25]

We undertook a systematic review:

1. to estimate the frequency of reported use of a DMC and the frequency of reported interim analysis and early stopping in a sample of recent pediatric clinical trials;

2. to assess the quality of the reporting on the various different DMC characteristics and on information about interim analysis and early termination in these trials.

## Methods

A systematic review was conducted using a sample of four general journals (*British Medical Journal*, *Journal of the American Medical Association*, *Lancet*, *New England Journal of Medicine*) and four pediatric journals (*Archives of Disease in Childhood*, *Archives of Pediatric and Adolescent Medicine*, *Journal of Pediatrics*, *Pediatrics*), published from January 2005 to December 2007. The included journals have a relevant impact factor in their field, and are known to publish pediatric trials in a broad range of conditions and age categories.

### Inclusion Criteria

Articles were included when they:

1. reported on a controlled clinical trial, randomized or quasi-randomized, investigating any type of therapeutic intervention (i.e. drug, procedure, behavioral, health strategy, or other) and including, but not necessarily confined to, participants between 0-18 years;

2. reported on the use of a DMC or interim analysis or early trial termination.

### Literature Search and Study Selection

Two searches were performed by one of the authors (R.F.). A Medline abstract search was used to identify the total number of pediatric trials for each journal, in order to obtain a denominator for frequency calculations. We used Medline's filters publication type "randomized controlled trial" and age category "0-18 years", and limited results to the specific journals and period under study. A separate full-text search was performed of the eight journals for the designated period of time using three full-text databases (*HighWire Press*, *OVID*, *ScienceDirect*). The search strategy for this full text search was adopted from Sydes et al and Montori et al[11,15] The resulting list of search terms, aimed at a high sensitivity, includes variations on terms related to DMC, interim analysis and early termination. For general journals, we added terms to identify trials with participants within the pediatric age range, from previously validated search strategies[26] The complete list of terms and the search strategy can be obtained from the corresponding author.

Titles and abstracts of the full-text search citation results were screened by one author (R.F.) for eligibility criteria. Two reviewers (R.F. and J.L.) subsequently screened the full text articles of included citations independently to confirm inclusion appropriateness. Any discrepancies were resolved through discussion until there was consensus. Motives for exclusion were noted and all search results were kept in database form.

### Data Extraction

Information was extracted from each included study report independently by two authors (R.F., J.L.) using data collection forms that can be obtained upon request from the corresponding author. The items included general trial characteristics, i.e. trial design, funding, centres and setting, details on the disease (based on the International Classification of Diseases, 10^th ^edition), age of participants, trial interventions, outcomes, planned and actual sample size, recruitment, intervention and follow-up time[27] Risks of bias in terms of substandard random sequence generation, allocation concealment, blinding and completeness of outcome data were assessed using the latest Cochrane Handbook criteria (v5, 2008), and disagreements were resolved by consensus[28]

There are currently only limited reporting standards for DMC related activities. Therefore, based on current literature, we defined *a priori *a set of parameters for data collection regarding DMC characteristics, interim analysis and early termination. For DMC characteristics and interim analysis, we consulted the book on Data Monitoring Committees by Ellenberg et al, and the report of the DAMOCLES group [1,2]. The latter presents empirical evidence and a set of recommendations on various issues related to DMCs' policies, structure, decision-making and reporting arrangements[3] We also included the CONSORT statement item on interim analysis[19] Early termination parameters were obtained from recent reviews addressing the implications of stopping trials early[11,13] For all three topics there appears to be consensus in the literature on best practices for some of the parameters, but many others remain controversial[2,7,12,14,29] Some items were not reported in the main report of the study, but in supplementary publications (e.g. online appendix, design/protocol papers). If these were referred to in the main paper, we included them in this review.

### Data Analysis

We performed a descriptive analysis of collected data, stratifying results by type of journal (i.e. whether general or pediatric). All data analyses were performed using SPSS (version 14; http://www.spss.com).

## Results

### Search Results and Prevalence of Reporting

**Figure 1:**
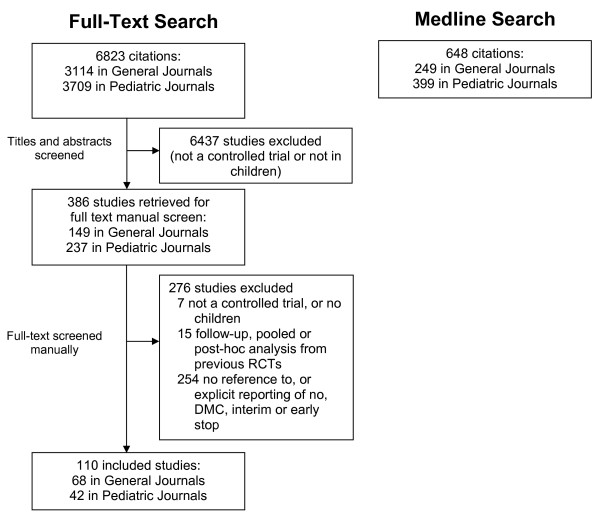
**Flow of citations identified through screening for pediatric trials reporting DMC, interim analysis or early termination**. Full-text databases search for DMC, interim analysis or early termination and Medline search for controlled pediatric trials, using Medline's filters.

**Figure 2:**
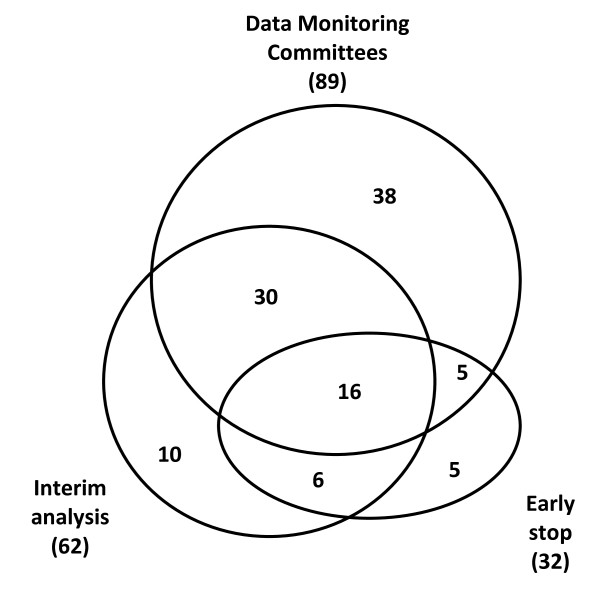
**Venn diagram summarizing the overlap in reporting on *Data Monitoring Committees*, *Interim Analysis *and *Early Termination *(n = 110)**. Number within each circle is the total number of articles which reported on these items, in isolation or combination.

Our search using Medline filters identified 648 controlled trials including pediatric subjects (249 from general journals and 399 from pediatric journals). The full-text search for DMC, interim and early termination terms identified a total of 6,823 potentially relevant records, which were screened for inclusion criteria. A flow diagram showing the yield of both searches is shown in Figure 1. Overall 110/648 trials (17%) reported on either, or DMC, interim analysis, or early termination, and were included in this review (Table 1 and Figure 2) [30-139] Reporting on these topics was more frequent in general journals (27%, 68/249) than in pediatric journals (11%, 42/399). DMCs were reported in 81% (89/110) of included trials, of which 52% (46/89) also referred to interim analysis. Of 62/110 (56%) trials that explicitly mentioned interim analysis, 74% (46/62) also reported use of a DMC. Early termination of a trial was reported in 32/110 (29%) articles, of which 21 (66%) reported on a DMC and 22 (69%) referred to interim analysis.

**Table 1:** Reporting of Data Monitoring Committees (DMCs) or Interim Analysis or Early Termination: Data on Included Randomized Controlled Trials (RCTs), and Total Number of Published RCTs, per Journal, from 2005 to 2007*

Journal	RCTs Reporting DMC or Interim/Total RCTs	RCTs Reporting Early Termination/Total RCTs
General Journals		
*BMJ*	3/62 (5%)	1/62 (2%)
*JAMA*	11/24 (46%)	1/24 (4%)
*Lancet*	23/83 (28%)	9/83 (11%)
*N Engl J Med*	31/80 (39%)	3/80 (4%)

Total	**68/249 (27%)**	**14/249 (6%)**

Pediatric Journals		
*Arch Dis Child*	2/37 (5%)	2/37 (5%)
*Arch Ped Adolesc Med*	4/61 (7%)	2/61 (3%)
*J Pediatr*	7/88 (8%)	3/88 (3%)
*Pediatrics*	24/213 (11%)	11/213 (5%)

Total	**37/399 (9%)**	**18/399 (5%)**

*The denominator refers to the total number of pediatric trials for each journal during the studied time period, which was obtained using Medline's filters (publication type "randomized controlled trial" and age category "0-18 years").

### Trial Characteristics and Risk of Bias

The main characteristics of the included trials are summarized in Table 2. Most studies were designed as parallel, superiority trials (87%, 96/110). Two-arm, drug treatment studies were predominant (85%, 93/110, and 73%, 80/110, respectively). A placebo group was included in 48% (53/110) of trials, while the others used an active comparator. The majority of trials were multicentre, and in 80/110 (73%) North American or European/Russian centres were included. Outcomes included mortality in 51% (56/110) of trials. Studies published in pediatric journals most frequently included neonates and addressed neonatal conditions (52%, 22/42), in intensive care settings (50%, 21/42). The median sample size in these journals was 136, and 60% (25/42) included less than 200 participants. The sample sizes included in trials reported in general journals were substantially larger (median 601 participants). Studies published in general journals more frequently included adolescent or adult participants (51%, 35/68, and 41%, 28/68, respectively), and the most common diseases under study were infections (44%, 30/68). Planned sample size calculations were reported in most trials (84%, 92/110). Where reported, time to primary outcome was often less than 12 months (81%, 69/85), and the overall median recruitment phase lasted 30 months.

**Table 2:** Characteristics of the 110 Trials Included in this Review, Stratified by Type of Journal

Parameter	Pediatric Journals (n = 42)	General Journals (n = 68)
**Design**		
Parallel	40 (95)	68 (100)
Cross-Over	2 (5)	0
**Factorial Trial**	0	7 (10)
**Cluster Trial**	3 (7)	3 (4)
**Noninferiority or Equivalence Trial**	4 (10)	8 (12)
**Centre^†^**		
Multicentre	26 (62)	58 (85)
Participants in more than one country	8 (19)	21 (30)
**Funding**		
Pharma only	10 (24)	15 (22)
Pharma and Institutional	3 (7)	9 (13)
Institutional only	24 (57)	44 (65)
Unclear	5 (12)	0
**Participants^†^**		
Neonates	23 (55)	17 (25)
Infants/Toddlers (<24 m)	11 (26)	27 (40)
Child (2-11 y)	14 (33)	34 (50)
Adolescents (11-18 y)	9 (24)	35 (51)
Adults	1 (2)	28 (41)
Category of Main Disease*		
Neonatal	22 (52)	9 (13)
Endocrine, nutritional and metabolic	6 (14)	3 (4)
Infections	2 (5)	16 (23)
Vaccine trials	3 (7)	14 (20)
Respiratory	3 (7)	7 (10)
Cancer	0	5 (7)
Other	6 (14)	14 (21)
**Setting**		
Outpatient	16 (38)	40 (59)
Inpatient	5 (12)	12 (18)
Intensive Care	21 (50)	16 (23)
**Type of Interventions**		
Drug	24 (57)	56 (82)
Invasive	6 (14)	5 (7)
Other	12 (29)	7 (10)
**Number of study groups**		
2 arms	38 (91)	55 (81)
>2 arms	4 (9)	13 (19)
**Use of Placebo or Sham arm**	18 (43)	35 (52)
**Duration of Intervention**		
Adequately reported	36 (86)	67 (97)
<1 month	23/36 (64)	35/67 (52)
1-12 months	11/36 (31)	21/67 (31)
>12 months	2/36 (5)	11/67 (17)
**Planned Power Calculation**	31 (74)	61 (90)
**Number of Participants**		
Median [IQR]^#^	136 [50-400]	601 [215-2360]
<50	11 (26)	0
50-200	14 (33)	15 (22)
200-1000	9 (21)	25 (37)
>1000	8 (19)	28 (41)
**Survival Outcome (primary or secondary)**	19 (45)	37 (54)
**Time to Primary Outcome**		
Adequately reported	28 (67)	57 (84)
<1 month	15/28 (54)	15/57 (26)
1-12 months	11/28 (39)	28/57 (49)
>12 months	2/28 (7)	14/57 (25)
**Recruitment Length**		
Adequately Reported	35 (83)	65 (96)
Median [IQR] months^#^	30 [21-38]	29 [22-48]

N (%) unless otherwise stated

†Each category is coded separately, so total may either exceed or not add up to 100%.

*Adapted from the ICD-10 Disease Categories

#IQR: Inter Quartile Range

Risk of bias assessments are shown in Table 3. We found average to high levels of adequacy in all parameters.

**Table 3:** Risk of Bias Assessments of the 110 Trials Included in this Review, Stratified by Type of Journal

Parameter	Pediatric Journals (n = 42)	General Journals (n = 68)
**Risk of Bias^†^**		
Adequate Sequence Generation	29 (69)	53 (78)
Adequate Concealment of Allocation	23 (55)	50 (74)
Adequate Blinding of Participants and Providers	21 (50)	34 (50)
Adequate Blinding of Outcome Assessment	25 (60)	53 (78)
Adequate Handling of Missing Data	30 (72)	53 (78)

N (%)

†Each category is coded separately, so total may either exceed or not add up to 100%.

### Reporting of DMCs and Interim Analysis

DMC nomenclature varied, and we identified 16 different expressions in use. "Data Safety Monitoring Board" (or similar, with conjunctions) was the most frequent (58%, 52/89), followed by "Data Safety Monitoring Committee" (16%, 14/89) and "Data Monitoring Committee" (7%, 6/89). The terms "external" or "independent" were added in 6% (5/89) and 30% (27/89), respectively. The remaining expressions included variations on the previously presented terms ("safety", "monitoring", "committee"), and the additional keywords "ethics" (n = 3), "monitor", and "group" (both n = 1). Authors sometimes used different expressions for DMC in the same paper[39,90,122,127] In some cases, the nomenclature and lack of additional information raised doubts as to whether the group in question was a DMC, particularly when dubious terms were used (e.g. "consultant") or there was a reference to other monitoring groups ("ethics", Independent Review Board, Outcome Adjudication Committee)[40,57,67,75,107,127] Additionally, in some papers we identified reports of an apparent role of the DMC in assessing or verifying outcome diagnoses[111,134]

In most papers DMCs and interim analyses were reported in the Methods section of the paper (89%, 93/105), sometimes in the Results (20%, 21/105) or in the Discussion (12%, 13/105) (Table 4). In one paper, interim analyses themselves were the main results[106] If a DMC was reported in the Acknowledgements section (66%, 69/105), the identity of the DMC members was always disclosed. In a few papers published in general journals other sources were referred to for additional details on DMC and/or interim analysis. These included appendices and online supplementary material on the journal website, trial design/protocol papers published in other journals, or trial or group dedicated websites[44,45,116,117,123,124]

**Table 4:** Reporting of DMC and Interim Analysis Parameters in trials that mentioned DMCs or Interim Analysis-Section of paper (n = 105)

Parameter	Pediatric Journals (n = 37)	General Journals (n = 68)
**Section of paper where DMC and/or interim analysis were reported^†^**		
Title/Abstract	4 (11)	6 (9)
Introduction	0	0
Methods	30 (81)	63 (93)
Results	13 (35)	8 (12)
Discussion	6 (16)	7 (10)
Acknowledgements	15 (41)	54 (79)
Online or other papers	0	7 (10)

N (%)

†Does not equal 100%, as some reports/articles contained data in more than one category

Tables 5 and 6 show the reporting of DMC and interim analysis parameters in the included papers. The most frequently reported items in each category were the identity of DMC members (75%, 67/89) and the number of interim analyses performed (84%, 52/62), respectively. The affiliation of DMC members was reported infrequently (28%, 25/89), as was their independence from industry sponsors (37%, 33/89). The least reported items in each category were vigilance of trial conduct parameters beside safety/efficacy outcome monitoring (19%, 17/89), and the adjustment of results for interim analysis (31%, 19/62).

**Table 5:** Reporting of details on DMCs in trials that mentioned DMCs (n = 89)

Parameter	Pediatric Journals (n = 26)	General Journals (n = 63)
**Members Identity**		
Reported	16 (62)	51 (81)
Unclear or Not Reported	10 (38)	12 (19)
**Member Affiliations**		
Reported	6 (23)	19 (30)
Unclear or Not Reported	20 (77)	44 (70)
**Independence**		
Reported	10 (39)	23 (37)
Unclear or Not Reported	16 (61)	40 (63)
**Monitored outcomes**		
Reported	11 (42)	39 (62)
Unclear or Not Reported	15 (58)	24 (38)
**Vigilance of other trial conduct parameters**		
Reported	5 (9)	12 (19)
Unclear or Not Reported	21 (81)	51 (81)
**Blinding to Primary Outcome Data**		
Reported	7 (27)	18 (29)
Unclear or Not Reported	19 (73)	45 (71)
**Predefined Stopping Guidelines**		
Reported	5 (19)	18 (29)
Unclear or Not Reported	21 (81)	45 (71)

N (%)

**Table 6:** Reporting of Interim Analysis Parameters in trials that mentioned Interim Analysis (n = 62)

Parameter	Pediatric Journals (n = 22)	General Journals (n = 40)
**Number of formal interim analyses planned**		
Reported	8 (36)	23 (58)
Unclear or Not Reported	14 (64)	17 (42)
**Number of formal interim analyses performed**		
Reported	19 (86)	33 (83)
Unclear or Not Reported	3 (14)	7 (17)
**Parameter defining timing of interim**		
Reported	18 (82)	30 (75)
Unclear or Not Reported	4 (18)	10 (25)
**Outcomes on which interim analyses were performed**		
Reported	13 (59)	32 (80)
Unclear or Not Reported	9 (41)	8 (20)
**Statistical monitoring methods specified**		
Reported	11 (50)	22 (55)
Unclear or Not Reported	11 (50)	18 (45)
**Adjustment of results for interim analysis** **(p value, CI or estimate)**		
Reported	2 (9)	17 (43)
Unclear or Not Reported	20 (91)	23 (57)

N (%)

No article adequately reported on all DMC parameters, i.e. members' identity and affiliations, independence, monitored outcomes, vigilance of other trial conduct items, blinding to outcome data and existence of predefined stopping guidelines (Table 5). Nine papers (15%) reported all interim analysis details, i.e. number of analyses planned and performed, parameter defining timing of these analyses, statistical methods used and outcomes to which they were applied, and adjustment of results (Table 6). We found two pairs of trials conducted by the same group of researchers, published in different journals, with distinct DMC nomenclature and reporting of most parameters[73,77,100,119] We also identified some discrepancies between protocols published as supplementary material and the papers' reports of DMC activities and interim analysis, e.g. reporting of one but not all statistical stopping rules [117,124].

Changes in protocol, most often sample size readjustments during the trial, were reported in 18% (20/110). The DMC was reported to have been involved in these decisions in 10 (50%) of these trials.

### Reporting of Early Terminated Trials

The frequency of reported early terminated trials in this sample was 21% (14/68) in general journals and 43% (18/42) in pediatric journals. Four of these trials terminated one or more arms of the trial but proceeded enrolment for the remaining interventions. DMCs and interim analysis were mentioned in 21/32 (66%) and 22/32 (69%) of early terminated trials, respectively. Reporting of parameters related to early termination is presented in Table 7. Only 28% (9/32) papers referred to early trial termination in the Title or Abstract, and 41% (13/32) addressed the implications of early termination in the Discussion section. In five trials (16%) the reason, e.g. harm, or rationale, e.g. statistical rules, for stopping early was not clear. Planned sample size was unclear or not reported in 13% (4/32) of trials, and the majority did not state predefined stopping guidelines (69%, 22/32). Statistical monitoring methods were not reported in 47% papers (15/32), while 44% (14/32) did not state the outcomes under monitoring or analysis. Only five papers (16%) reported that the final results were adjusted because of the interim analysis, e.g. by using an alpha spending approach, four (13%) of which reported all interim analysis parameters presented in Table 6. Overall, only two trials (6%) reported all relevant methodological elements for interpretation of results from early terminated trials, i.e. planned sample size, predefined stopping guidelines, statistical monitoring methods used, rationale for early termination, timing of early stop (i.e. whether trial termination was determined by one of the interim analysis, and which parameter determined the timing of this interim), whether results were adjusted, and recommendation of DMC.

**Table 7:** Reporting of Early Termination Parameters in Trials Terminated Early (n = 32)^†^

Parameter	Pediatric Journals (n = 18)	General Journals (n = 14)
Motive(s) for termination*		
Adequately reported	18 (100)	12 (86)
Terminated for efficacy	3 (17)	3 (21)
Terminated for harm	2 (11)	4 (29)
Terminated for futility	3 (17)	4 (29)
Other reasons reported (stand-alone or additional)	13 (72)	2 (14)
**Rationale for termination** **(i.e. stopping guidelines/rules)**		
Reported	15 (73)	13 (93)
Unclear or Not Reported	3 (17)	1 (7)
**DMC role in early termination**		
Reported	3 (17)	12 (86)
Unclear or Not Reported	15 (83)	2 (14)
**Timing of early termination^#^**		
Reported	8 (44)	8 (57)
Unclear or Not Reported	10 (56)	6 (43)
**Adjustment of results for interim analysis (p value, CI or estimate)**		
Reported	2 (11)	8 (57)
Unclear or Not Reported	16 (89)	6 (43)
**Section of paper where reference to early termination was reported**		
Title/Abstract	4 (22)	5 (36)
Methods	14 (78)	14 (100)
Results	13 (72)	6 (43)
Discussion	10 (56)	3 (21)

N (%)

†refers to trials with one or more arms terminated before planned

*Does not equal 100%, as some reports/articles contained data in more than one category

#reporting was considered adequate when it was clear that trial termination was determined by one of the interim analyses, and on which parameter the timing of this interim analysis was based

## Discussion

Our study shows that the reporting of parameters related to DMCs, interim analysis and early termination in pediatric trial reports is heterogeneous and incomplete. This result is consistent with recent reviews of trials from various settings outside child health[8,11,13,15-18] The CONSORT statement for standards of reporting clinical trials includes an item on interim analysis, but no details on DMCs or early termination[19] We propose a minimal set of reporting parameters that will allow the reader of a trial report to assess their implications and the validity of trial results (Table 8). Also, we propose to update the CONSORT statement accordingly. Below we discuss the various components of this minimal set of reporting parameters, as they appear - from our study - to be relevant for the correct design, conduct and reporting of pediatric trials.

**Table 8:** Recommendations for a minimal set of parameters to be reported and included in trial registries regarding DMC activities, Interim Analysis and Early Stopping^† ^*

Data Monitoring Committees and Interim Analysis*
**Terminology**
Use of the standard nomenclature "Data Monitoring Committee"
**Composition of the DMC**
Members' name, affiliation and training
Independence status from research team and sponsor
**Tasks of the DMC**
Whether the DMC reviewed and accepted the protocol before the start of the trial
Main roles (e.g. monitoring of safety and/or efficacy), and explicit definition of which outcomes where analyzed^#^
Any additional roles (e.g. monitoring recruitment, quality assurance)
To which outcome(s) was the DMC blinded or unblinded
**Interim Analysis and Statistical Monitoring Methods**
Whether the protocol included a predefined statistical monitoring plan
Number of planned interim analyses
Timing of planned interim analyses and parameter defining timing (i.e. participants or person-time recruitment, number of endpoints, ad hoc time interval)
Type of analysis planned (i.e. efficacy, harm, futility, and/or sample size adjustment), specific statistical methods used (with references and uniform terminology), description of boundaries (i.e. their symmetry, p-value/confidence interval, and adjustment, if applicable), and outcome(s) to which they were applied (i.e. primary/secondary, any subgroup analysis)
Any formal predefined stopping rules, to which outcome(s) did they apply, and whether they included statistical boundaries and/or other considerations
Whether the statistical monitoring plan was completed as planned; if not, which changes were performed, and their rationale
Adjustment for multiple analysis in final results (i.e. reported p-values and/or confidence intervals)
**Recommendations to the sponsor/steering committee**
DMC recommendation regarding continuation or termination of the trial (with or without adjustments in protocol)
Rationale (i.e. statistical boundaries and/or other considerations)
Whether the sponsor followed the DMC's recommendations

**Early terminated trials**

Motive(s) for termination (e.g. efficacy, harm, futility, recruitment)
All previously stated items, particularly rationale for early termination (including predefined statistical monitoring plan, type of analysis, predefined stopping rules, and DMC recommendation), and adjustment for multiple analysis and early termination in final results
Timing of early termination i.e which of the interim analyses led to trial termination, and on which parameter the timing of this interim analysis was based (e.g. number of participants enrolled, predefined number of endpoints)
Planned and final sample size
Total number of events after which the trial was terminated, including definition of these events
Discussion of implications of early termination (i.e. concerning type I and II errors)
Report early termination in the abstract of the paper

†These recommendations are for main reports; further details could be available using other modes of publication (e.g. online appendices, trial design/protocol papers, web-based repositories), to which the report should refer to; planned items of this minimal set of parameters should be included in prospective trial registries.

*Based on the book by Ellenberg et al, and the report of the DAMOCLES group [1],[2].

#Particularly regarding safety-whether it included adverse events and/or main efficacy outcomes.

### Use of DMCs in Pediatric Clinical Trials

In a review of pediatric trials published until 2002, only 2% were found to refer to safety committees[23] Reporting of DMCs was more frequent in our - more recent - sample of pediatric studies, but our results are comparable to those in other recent reviews covering all age ranges[15,18] This suggests a recent increased use of DMCs, but other relevant factors probably account for this difference. First, bias may have arisen from using a selected sample of high-impact journals, as reporting of DMC may differ according to publication practices and trial results. Second, differences observed between general and pediatric journals in the current review may be confounded, as we can expect trials published in high-impact general medical journals to have characteristics which are more frequently associated with the use of a DMC (e.g. large samples, new interventions).

Deciding whether a clinical trial actually needs a DMC involves consideration of the safety of the interventions under study, the type of outcomes, and which population is included, among other items[1,4,5,7] Regulators recommend considering DMC involvement in the presence of vulnerable participants, such as children, but these are mere recommendations without obligations and no further operational guidance is given[4,5] We found that DMCs are being reported in trials with participants of different age ranges and a wide range of conditions. These include serious, potentially fatal diseases (e.g. leukemia, bronchopulmonary dysplasia), but also minor conditions (e.g. caries, pharyngitis). Pediatric trials most often have specific ethical, safety and design issues that make a DMC indispensible, and, therefore, various pediatric specialty committees recommend their use[24,140-143] Given the results of this study, we feel there is a need for a standard in the field of pediatric trials, including operational criteria for the establishment, roles and conduct of DMCs. We recommend to consider DMCs at least for pediatric trials concerning serious conditions and in which the recruitment time is long compared to the follow-up time for individual participants, which makes interim decisions with consequences for further inclusion possible.

### Alternative Terminology

The use of different expressions to denote DMCs may be attributed to local or national preferences, as well as to discrepancies in DMC-related guidance documents from various sponsors and institutions[2,7,15] Standardized nomenclature is necessary in order to distinguish DMCs from on site monitoring by trial centres, and from other trial oversight groups (e.g. Institutional Review Boards, Ethics Committees)[5] It is particularly relevant to recognize each group's distinct and non-overlapping role in ensuring the validity of the trial[144] To avoid confusion, we recommend the use of a single expression "Data Monitoring Committee", as suggested by the DAMOCLES group (see Table 8) [3]

### Composition of the DMC

The impact of DMCs' recommendations in trials calls for transparency and accountability of its members. In our review DMC members' identities were commonly reported, but not their affiliations, nor their independence. Independence is particularly relevant and relates to real or perceived conflicts of interest of each DMC member with regard to the sponsor, the intervention under study, and the regulator[1,4,5,7]

Three main models for the composition of a DMC have been proposed previously, with varying degrees of independence from the research team and the sponsor[1,7,145] First, trials may have an "internal" DMC with non-independent members, e.g. the principal investigator, or they may have a monitoring plan without a formal DMC. A second option is to include only members not involved in the trial, except for the trial statistician, who acts as the DMC statistician. Third, all DMC members, including the statistician, are independent[7] In any case, appointment and funding by sponsors may affect this independence[4,5] Knowing how a DMC was established is paramount for accepting the integrity and for the credibility of this DMC's decisions. Trial reports therefore should identify members and their affiliations, and disclosure of conflicts of interest should be made available. They should also make a clear statement about their independence, possibly with an operational definition (see Table 8).

### Tasks of the DMC, Monitoring and Interim Analysis

Our findings show inadequate reporting on which roles were taken by a DMC and how they were performed. Ellenberg et al states that monitoring safety, efficacy and other trial conduct parameters are among main DMC activities[1] Activities should be clearly agreed upon and defined in advance by DMC members, trial sponsors and investigators, preferably using a "charter"[3]

Outcome data may be monitored in a number of ways, particularly safety data, which can include either or both adverse events and other safety/efficacy outcomes[146] There is controversy as to the blinding of DMCs[7] Reporting of these procedural aspects is needed to allow clinicians to judge decision-making (see Table 8).

We found discrepancies in reporting of the statistical monitoring plan and the use of interim analysis. Ellenberg et al highlight the relevance of predefined stopping guidelines, including a prudent use of statistical boundaries[1] Indeed, performing multiple looks at data through interim analyses without correction can lead to multiplicity problems, with increased risk of type I error[147] A number of statistical monitoring methods have been developed to overcome this issue, among which the frequentist, Bayesian and likelihood approaches[2] As each statistical method has different assumptions and implications, reporting should be clear and unequivocal[2,10,29,148] Additionally, there are differences between analyses aimed at efficacy, harm or futility, as well as the possible use of alternative trial designs (e.g. adaptive)[149] This is compounded by a nomenclature which is often inconsistent and overlapping (e.g. "adaptive designs" vs. "sample size reviews")[149] Clear reporting should allow readers to identify these methods and their impact of on type I and II error risks (see Table 8).

### Early Terminated Trials

We found similar deficiencies in reporting on early termination as were reported previously in other samples of trials[11,16,17] A significant number of early terminated trials did not report on the existence of a DMC or on its role in early stopping. This raises doubts as to which trial oversight group recommended termination, and whether this recommendation was issued independently from sponsor and principal investigators.

Understanding the rationale for early termination is paramount to a correct assessment of the validity of trial results. This implies knowing which, if any, statistical monitoring methods and stopping rules or guidelines were used, for which outcome (whether primary or secondary), and at which stage of the trial[11,13] It may be relevant in some cases to check if the results were corrected for multiplicity[147] The ongoing controversies regarding early termination highlight the need for transparent reporting of these items (see Table 8).

Risks of early termination for benefit include implausibly large treatment effects[11,12,150] Additionally, futility analyses and protocol changes, like sample size reviews and adaptive designs, may interfere with the overall power of the trial[10,148,149] The majority of authors did not discuss these implications of early termination in their report. Because readers often base their assessment of a trial solely on information in the abstract, this should clearly report early termination and its rationale, as recommended in the latest CONSORT statement extension for journal and conference abstracts[151]

### Strengths and Limitations of this study

Our full-text search strategy allowed a thorough review of all published content in the selected journals. This strategy was chosen since the reporting of DMCs, interim analysis and early termination is not indexed in Medline records, and it is often not reported in the abstract. We opted to use Medline's filters to identify pediatric controlled trials, although we can expect some indexing inaccuracies.

We only analyzed published reports, and we did not contact authors to evaluate whether DMC and interim analysis were used but not reported. This will likely lead to an underestimation of their actual use, as previously observed with the reporting of other trial methodology issues[152] Reports of studies in which DMCs had an active role, particularly when early termination decisions were taken, may be also more likely to report details on their activities. A relatively large proportion of trials from general journals included adult participants. Results from these trials may not apply to studies that were designed to include children exclusively, as these trials may differ for example regarding the presence of a DMC and its tasks and procedures.

The sample of trials for our review was chosen from recent volumes to have an estimate of current practice. Most of these trials were, however, designed, conducted and published before the latest standards for DMC use (i.e. DAMOCLES) were published. We expect that DAMOCLES will have a future impact on trial design, conduct, and reporting[3]

There was a moderate to high level of adequacy in the reporting of risk of bias parameters indicating good adherence to the CONSORT statement on these items. These results show that there is an apparent shortcoming in the reporting of DMCs, interim analyses and early termination, even in trials which appeared well designed and reported in other aspects. Until CONSORT requires more information of DMC activities, we expect that reporting will remain poor. Therefore, we propose to update the CONSORT statement according to the recommendations described below.

### Recommendations for Reporting of DMC, Interim Analysis and Early Termination (Table 8)

We agree with the EQUATOR Group that continued efforts are needed to improve reporting quality, by updating and disseminating current standards[153] With the current increase of clinical trials in child health, and given our results, we feel this has become urgent in this field. We propose a list of reporting items on DMC terminology, composition, tasks, and recommendations, on statistical monitoring methods and interim analysis, and on early termination parameters. This could be considered in the next CONSORT revision. Space limitations should not preclude transparent reporting, given the current possibilities of online publishing. Discussion should be started on the inclusion of this minimal set of parameters in design/protocol papers and trial registries[154,155]

## Conclusion

Reporting of DMC activities, interim analysis results and early termination of pediatric trials is incomplete and heterogeneous. New standards for the reporting of these parameters are necessary in order to evaluate their impact on the validity of trial results.

## Abbreviations

*Arch Dis Child: Archives of Disease in Childhood; Arch Ped Adolesc Med: Archives of Pediatric and Adolescent Medicine; BMJ: British Medical Journal; *DMC: Data Monitoring Committee; *JAMA: Journal of the American Medical Association; J Pediatr: Journal of Pediatrics; N Engl J Med: New England Journal of Medicine; *RCT: Randomized Clinical Trial.

## Competing interests

The authors declare that they have no competing interests.

## Authors' contributions

All authors contributed towards the conception and design of the study and the interpretation of the data. They also read, edited and approved the final manuscript. RF performed the full-text search. RF and JL identified relevant studies and participated in the data extraction and data analysis. RF drafted the manuscript.

## Pre-publication history

The pre-publication history for this paper can be accessed here:

http://www.biomedcentral.com/1471-2431/9/77/prepub
